# The relationship between activities of daily living and suicidal ideation among Chinese rural older adults: a multiple mediation model through sleep quality and psychological distress

**DOI:** 10.18632/aging.103857

**Published:** 2020-11-17

**Authors:** Dan Zhao, Jie Li, Wenting Hao, Yemin Yuan, Caiting Yu, Zhengyue Jing, Yi Wang, Peipei Fu, Chengchao Zhou

**Affiliations:** 1 School of Public Health, Cheeloo College of Medicine, Shandong University, Jinan 250012, China; 2 School of Health Care Management, Cheeloo College of Medicine, Shandong University, Jinan 250012, China; 3 NHC Key Laboratory of Health Economics and Policy Research, Shandong University, Jinan 250012, China

**Keywords:** physical functioning, suicidality, sleep disturbances, psychological well-being, multiple mediation

## Abstract

Few studies clarified the mechanisms underlying the relationship between activities of daily living and suicidal ideation among older adults. This study aimed to explore the multiple mediating roles of sleep quality and psychological distress between this relationship. A total of 3,243 rural older adults were included. Multiple mediation analysis was performed using Mplus 8.3. Activities of daily living was found to directly affect suicidal ideation (β=0.092, 95% CI=0.043−0.140) and through three significantly mediation pathways: (1) the path through sleep quality (β=0.019, 95% CI=0.007−0.031), which accounted for 9.79 % of the total effect; (2) the path through psychological distress (β=0.049, 95% CI=0.036−0.063), which accounted for 25.26 % of the total effect; (3) the path through sleep quality and psychological distress (β=0.034, 95% CI=0.026−0.042), which accounted for 17.53 % of the total effect. The total mediating effect was 52.58%. Attention should be paid to sleep quality and mental health among the Chinese rural older adults with activities of daily living limitation. For early detection and prevention of suicidal ideation, it is necessary to take sleep-based and positive psychological interventions for older adults with activities of daily living limitation.

## INTRODUCTION

Suicide is a global public health issue. In China, the number of suicides was close to 140,000, indicating an annual age-standardized suicide rate of 8.0 per 100,000 population [1]. China’s suicide rates among older adults have remained higher in rural than in urban areas [2]. Suicide prevention of rural older adults is a public health priority and urgently needs to be put on the agenda in China. Suicidal ideation (SI), the first step of suicide based on the Three-Step Theory (3ST) of the “ideation-to-action” framework, has been identified as the most sensitive predictor of suicidal behavior [3, 4]. The prevalence of SI was relatively high among Chinese older adults, ranging from 2.2% to 21.5% [5]. Identifying risk factors of SI is critical to preventing suicide.

Physical dysfunction has been demonstrated to be one of the most common risk factors associated with SI among older adults [6]. Activities of daily living (ADL) was an important indicator of the physical disability among the elderly, which is found to be a significant risk factor for suicide [7]. It can not only increase the possibility of SI, but also promote the transition from SI to suicidal behavior. A study indicated that ADL disability was associated with SI among Chinese rural older adults [8]. Although an association was found between ADL and SI, the potential factors underlying this association are poorly understood. Exploring the mediation pathways between ADL and SI is helpful to find more effective ways to provide useful information for early detection and prevention of SI. It can also provide a significant theoretical framework for clinical practice and public health work in the suicide prevention among older adults.

A previous research has indicated that psychological distress was a predictor of first onset and persistence of SI [9]. A path analysis among Chinese older adults with hypertension demonstrated that psychological distress had a significant mediating role in the association between related factors and SI [10]. Another study found that psychological distress was significantly correlated with ADL among Chinese older adults [11]. Recently, Zhu et al. found that psychological distress played a mediating role between ADL and SI [12]. Based on the above analysis, psychological distress might be a mediator for ADL and SI.

Sleep quality was closely related to the physical and mental health of individuals. Several components of sleep quality were found to be associated with increased suicide risk, such as cough or snore loudly [13–15]. Some other studies among older people suggested that ADL disability was significantly associated with sleep quality [16]. The aforementioned results indicated that older adults with significant ADL disability were more likely to have poor sleep quality. These findings demonstrated that sleep quality was related to both physical dysfunction and SI. However, there is still a lack of empirical studies that directly investigate sleep quality as a mediator between ADL and SI.

A study in Australia demonstrated that adults with comorbid psychological distress increased the risk of poor sleep quality [17]. Among Chinese older adults, a study showed that there was also a significant correlation between sleep quality and psychological distress, the better the quality of sleep, the less the psychological distress [18]. Preliminary evidence suggested that the association between sleep problems and suicidal thoughts might function via psychological factors [19]. Another study in Japan also found that insomnia symptoms were positively associated with SI through the mediator of depressive symptoms [20]. From the above analysis, it is possible that sleep quality and psychological distress may act as serial mediators of the relationship between ADL and SI.

**Figure 1:**
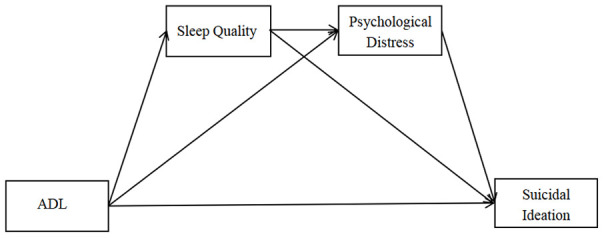
**Hypothesized model.**

However, to date, no studies have comprehensively explored the factors underlying the relationship between ADL and SI among the Chinese rural older adults. The present study aimed to explore the multiple mediating roles of sleep quality and psychological distress in the relationship between ADL and SI among Chinese rural older adults, so as to provide scientific evidence for SI prevention. Therefore, we proposed three hypotheses developing the hypothesized model of this study (Figure 1). First, it was hypothesized that ADL could mediate SI through sleep quality (H1). Second, it was hypothesized that psychological distress was a mediator for the association between ADL and SI (H2). Third, it was hypothesized that sleep quality and psychological distress could be the two chain mediators in the relationship between ADL and SI (H3).

## RESULTS

### Characteristics of participants

Of all respondents, 343 (10.6%) had SI. The mean (SD) scores of ADL for respondents with and without SI had significant difference (*Z*=-8.18, *P*<0.001). With regard to the psychological distress, the suicide ideators had higher level of psychological distress (*Z*=-17.48, *P*<0.001). The mean (SD) PSQI score of suicide ideators was higher than the counter part of the older adults (*Z*=-11.46, *P*<0.001). More details of the participants’ characteristics are shown in Table 1.

**Table 1:** Description and univariate analysis of suicidal ideation among the seniors in Shandong, China, 2019 (N=3,243).

**Characteristics**	**N (%)**	**Suicidal ideation**		$\chi^{2}\text{/}Z$	***P*-value**
		**No (%)**	**Yes (%)**		
Observations	3,243	2,900(89.4)	343(10.6)		
Gender				35.07	*<***0.001**
Male	1181(36.4)	1106(93.6)	75(6.4)		
Female	2062(63.6)	1794(87.0)	268(13.0)		
Age, years, M (P_25_, P_75_)	70 (65,74)	70 (65,74)	69 (65,74)	-1.68	0.092
Education				3.28	0.194
Illiterate	1354(41.7)	1198(88.5)	156(11.5)		
Primary school	1257(38.8)	1126(89.6)	131(10.4)		
Middle school or above	632(19.5)	576(91.1)	56(8.9)		
Marital status				0.53	0.513
Single^a^	828(25.5)	746(90.1)	82(9.9)		
Married	2415(74.5)	2154(89.2)	261(10.8)		
Living arrangement				3.01	0.083
Empty-nesters^b^	2654(81.8)	2385(89.9)	269(10.1)		
Non-empty-nesters	589(18.2)	515(87.4)	74(12.6)		
Employment status				34.46	*<***0.001**
Unemployed	1366(42.1)	1174(85.9)	192(14.1)		
Agricultural work	1617(49.9)	1480(91.5)	137(8.5)		
Non-agricultural work	260(8.0)	246(94.6)	14(5.4)		
Household income per capita^c (CNY)^				18.39	*<***0.001**
Q1	809(24.9)	693(85.7)	116(14.3)		
Q2	811(25.0)	734(90.5)	77(9.5)		
Q3	809(24.9)	725(89.6)	84(10.4)		
Q4	814(25.1)	748(91.9)	66(8.1)		
Cigarette smoking				18.78	*<***0.001**
No	2240(69.1)	1968(87.9)	272(12.1)		
Yes	1003(30.9)	932(92.9)	71(2.2)		
Alcohol drinking				15.92	*<***0.001**
No	2321(71.6)	2044(88.1)	277(11.9)		
Yes	922(28.4)	856(92.8)	66(7.2)		
Physical exercise				29.93	*<***0.001**
No	1580(48.7)	1365(86.4)	215(13.6)		
Yes	1003(30.9)	932(92.9)	71(2.2)		
ADL, M (P_25_, P_75_)	16 (14,18)	16 (14,18)	17 (15,20)	-8.18	*<***0.001**
Sleep quality,M (P_25_, P_75_)	7 (4,11)	7 (4,11)	11 (7,15)	-11.46	*<***0.001**
Psychological distress, M (P_25_, P_75_)	14 (10,21)	13 (10,20)	24 (18,30)	-17.48	*<***0.001**

*Notes:* ADL= activities of daily living; ^a^ Singles include those who are unmarried (38, 1.17%), divorced (12, 0.37%) and widowed (778, 23.99%); ^b^ the empty-nesters elderly refers to those elderly with no children or whose children have already left home, and they either live alone or with a spouse; ^c^ Q1 was the poorest and Q4 was the richest; ^CNY:^ the currency symbol assigned by International Organization for Standardization to China.

### Correlation between variables

The correlation matrix for key study variables is provided in Table 2. The correlation matrix for key study variables is provided in Table 2. ADL (*r*=0.144, *P*<0.001), sleep quality (*r*=0.201, P<0.01), and psychological distress (*r*=0.307, *P*<0.001) were all positively related to SI. The higher the scores, the more likely older adults have SI.

**Table 2:** Spearman correlation coefficients between key study variables (N=3,243).

**Variables**	**1**	**2**	**3**	**4**
1.Suicidal ideation	1.000			
2.ADL	0.144*	1.000		
3.Sleep quality	0.201*	0.181*	1.000	
4.Psychological distress	0.307*	0.175*	0.448*	1.000

*Notes:* ADL= activities of daily living; df =3,239; * *P*-value<0.001.

### Mediating effect analyses

**Figure 2:**
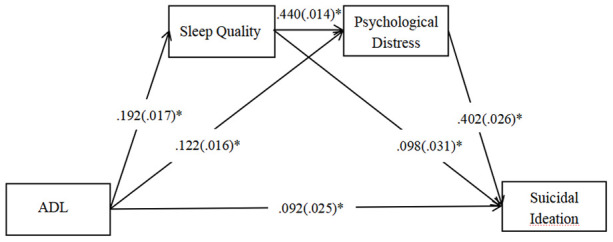
**A multiple mediation model of the association between ADL and suicidal ideation through sleep quality and psychological distress.** Standard error in the parentheses and path coefficients are shown. Note, Posterior Predictive *P*-value = 0.495, *P-value<0.001.

Figure 2 illustrates the mediation pathway model. The fit index (PP*P*=0.495) showed it was an excellent-fitting model. Path coefficients showed that all relationships in the model were significantly positive. After including the mediators of the sleep quality and psychological distress, the direct effect of ADL on SI was still significant. Therefore, the association between ADL and SI was achieved partly through these two mediators.

The total effect, direct effect and indirect effects are described in Table 3. Specifically, the standardized effect value of ADL on SI through sleep quality was 0.019, with the mediating effect of 9.79 %. The standardized effect value of ADL on SI through psychological distress was 0.049, with the mediating effect of 25.26 %. The standardized effect value of ADL on SI through sleep quality and psychological distress was 0.034, with the mediating effect of 17.53 %. The total mediating effect of ADL on SI was 52.58 %, which was stronger than the direct effect of ADL on SI.

**Table 3:** The standardized total, direct, and indirect effects of ADL on suicidal ideation with sleep quality and psychological distress as mediators (N=3,243).

**Model pathways**	***b***	**Posterior S.D.**	**95% C.I.**	**Mediating effect**
Total effect ADL→suicidal ideation	0.194*	0.025	(0.144,0.241)	100%
Direct effect ADL→suicidal ideation	0.092*	0.025	(0.043,0.140)	47.42%
Total indirect effect ADL→suicidal ideation	0.102*	0.009	(0.084,0.120)	52.58%
ADL→sleep quality→suicidal ideation	0.019*	0.006	(0.007,0.031)	9.79%
ADL→psychological distress→suicidal ideation	0.049*	0.007	(0.036,0.063)	25.26%
ADL→sleep quality→psychological distress→suicidal ideation	0.034*	0.004	(0.026,0.042)	17.53%

*Notes:* ADL=Activities of daily living; C.I.= Bayesian Credible Interval;

* *P*-value<0.001.

### Multivariate analysis

A multivariate logistic regression analysis was performed to determine the association of suicidal ideation and other key variables (see Supplementary Table 1). ADL (*P*<0.001), sleep quality (*P*< 0.001), and psychological distress (*P*<0.001) were significantly associated with SI. When we included controlling variables in the models, they were still associated with SI, respectively.

## DISCUSSION

The current study found that the prevalence of SI among rural older adults in Shandong Province was 10.6%. This was similar to another survey conducted in rural Shandong (11.0%) [12]. It was also close to the reported rates in the older US Chinese population (9.4%) [21]. However, it was much higher than that in rural Beijing (5.75%) [22] and urban Shanghai (3.1%), China [23]. Because of differences in domestic economic development levels and the advancement of urbanization, rural elderly people were more likely to have SI due to lack of health care and poor living conditions [24].

The present study found that ADL was positively associated with SI directly, and also indirectly, through sleep quality and psychological distress. The significant direct effect was consistent with previous studies [7, 8], which indicated that the higher the degree of physical dysfunction, the more likely the older people were to suffer from SI. If the older adults have ADL limitation, they are less likely to be able to take care of and support themselves. It may make them feel out of control and have negative thoughts such as SI. However, in this research, the total mediating effect was 52.58 %, which was stronger than the direct effect, revealing that our mediators were critical in explaining the association between ADL and SI.

Our findings showed that sleep quality mediated the relationship between ADL and SI with the mediating effect of 9.79 %. This study is the first to examine the mediated effect of sleep quality on the ADL-SI relationship. Therefore, hypothesis H1 was testable: older adults with ADL limitations are more likely to have SI through poor sleep quality. This was in accordance with previous studies revealing that increased functional disability was associated with increased sleep problems [16] and poor sleep quality was a risk factor for SI [15]. The prevalence of sleep disorders was higher among older adults. Study suggested that when physical function declines, the older adults are more likely to have sleep problems [25]. Older adults with dysfunction would have poor sleep quality, which may reduce their amount of activity and shorten the sleep awakening cycle. In addition, less activity led to a reduction in functional reserves that improved sleep quality. Holdaway et al. suggested that sleep quality exacerbated the association of reflective rumination or other negative thoughts with overall suicide risk and SI [26]. Other studies showed that sleep quality also played a mediating role on SI, such as the relationship between school climate and SI, problematic Internet use and suicidal behavior [27, 28].

Results from our study indicated that psychological distress was a mediator in the relationship between ADL and SI. Thus, hypothesis H2 was testable: older adults with ADL limitations are more likely to have SI through psychological distress. Psychological distress was responsible for 25.26 % of the ADL related to SI, which revealed that ADL moderated SI mainly through the psychological distress. Previous studies have already found that the psychological distress was positively correlated with SI [9, 29]. The higher the level of psychological distress, the more likely to the older adults suffer from SI. A study has shown that ADL disability promoted SI by increasing psychological distress among Chinese rural older adults [12]. Our study revealed that psychological distress played the largest mediating role in the association between ADL and SI. Possible reasons were ADL disability could increase physical pain and financial difficulties for older adults, leading to increasing negative emotions. When the older adults had difficulties in daily living, it was easy to produce the feeling of pain, anxiety and even desperation [30]. They may seek more help from others in their lives, causing themselves to feel guilty and useless, which seriously affected their mental health. In addition, because of social restrictions, they may not be able to relieve their psychological distress and eventually experience SI.

We also found that the relationship between ADL and SI was partially mediated through chain mediation by sleep quality and psychological distress with an effect size of 17.53 %. Thus, hypothesis H3 was testable: older adults with ADL limitations are more likely to have SI through poor sleep quality and psychological distress. The mediation effect of this path ranked second, which is relatively high. More attention should be paid to disabled older adults who have both psychological distress and sleep problems. According to the American Sleep Association, insomnia was the most common specific sleep disorder, adversely affecting people’s physical and mental health in many ways [31, 32]. Sleep problem was one of the most common secondary conditions in women with physical disabilities [33]. Besides, sleep quality had a significant influence on psychological distress, and it played a partially mediating role between interpersonal distress and psychological distress [34]. Preliminary evidence indicated that psychological factors mediated the association between sleep problems and SI through negative cognitive appraisals, social isolation and thwarted belongingness, and emotion regulation strategies [19]. Physically disabled older adults often experience significant sleep disturbance, which may be a risk factor for poor physical and emotional functioning, including increased anxiety and fear, ultimately increased the possibility to suffer from SI.

### Enlightenments and limitations

This study is the first to comprehensively explore the factors underlying the relationship between ADL and SI among the Chinese rural older adults, which is of theoretical and practical significance to provide useful information for early detection and prevention of SI. Firstly, the government should focus on the living conditions of disabled older people. The government can increase the subsidy for older adults with dysfunction. Local governments and community committees can also set up a tracking system for them and pay attention to their recovery. Secondly, attentions should be paid on the sleep quality of disabled older adults so as to develop appropriate health education in the community. Older people who have problems in sleep quality should see a doctor promptly. The third and most important point is to pay attention to the mental health of rural disabled older people. The government and community committees should establish rural psychological counseling rooms to regularly provide psychological counseling service for those at-risk older adults.

Several limitations of this study also need to be acknowledged. First, this study was a cross-sectional study. The directionality in the interrelationships between ADL impairment, sleep quality, psychological distress and suicidality remains unclear. But the relationships between variables were based on existing literature and theoretical support [8, 12, 15, 17, 19, 20]. In future research, these findings can provide the groundwork for alternative designs, such as longitudinal designs and interventional experiments for further verification. Second, using self-reported data to measure sleep quality was limited and it would be interesting to use sleep monitoring equipment or biological methods in the future. For the outcome variable, using a single item from the National Comorbidity Survey to assess SI was relatively crude, which would be remedied in the follow-up studies. Finally, this study was applicable to older adults in rural China, and other populations need to be verified in future studies.

In conclusion, this study explained the underlying factors between ADL and SI. The association between ADL and SI was achieved through the partial mediation of sleep quality and psychological distress. These findings imply a need to develop policies and pay attention to the sleep quality and mental health among Chinese rural disabled older adults, so as to develop early detection and early prevention of SI.

## MATERIALS AND METHODS

### Sample selection

This study was conducted in Shandong province from May to June in 2019. According to the National Bureau of Statistics in 2018, the population of Shandong Province ranked the second in China, breaking through the 100 million mark for the first time. The sample size calculation was performed by the following formula: $N=\frac{u_{\alpha /2}^{2}\times \pi (1-\pi )}{\delta^{2}}$ (*π*: expected prevalence) [35]. The prevalence of SI was 11.0% among Chinese rural older people according to previous studies [12], so in this study, *π* = 0.11, *u_α_*_/2_ = 1.96, δ = 0.1 *π* = 0.011, *α* = 0.05. We found that required sample size was 3,109. Taking into account the loss and refusal of interviews, the final sample size of the survey was determined to be 3,600.

China has a three-level local administrative division system at the provincial, county, and township levels. In order to facilitate management, the Chinese administrative system has formed a four-level system of provinces, cities, counties, and townships. Villages, the most fundamental rural unit, affiliated with township management, which corresponding to communities in urban areas. We used a three-stage stratified cluster sampling method to select participants. Firstly, all counties were divided into three groups according to the GDP per capita (2018), and then one county was randomly selected from each group. Three counties (Qufu, Laolin, Rushan) from three prefectural cities (Jining, Dezhou, Weihai) were selected as study sites. Secondly, analogously, five townships were randomly selected from each county and four sample villages were randomly selected from each township. Thirdly, 60 or more elderly families (the family with at least one elderly person aged 60 years and above) were investigated in each sample village. Finally, 15 townships and 60 villages were selected. In total, 3,600 questionnaires were distributed, and 3,243 valid questionnaires were included in this study, with a response rate of 90.05%.

### Data collection

Well-trained postgraduate students of Shandong University interviewed older adults face to face using a structured questionnaire. The inclusion criteria of the respondents were older adults aged 60 years and above who were informed about the content of this study and able to communicate. In order to obtain complete and accurate data, we excluded participants who are unable to communicate normally due to physical condition or some other reasons and unwilling to cooperate with interviews. To ensure the quality of the data, the quality supervisors checked carefully the completed questionnaires at the end of the interview on each day.

### Measures

### Sociodemographic characteristics

Sociodemographic characteristics included gender (male, female), age (years), education (illiteracy, primary school, middle school or above), marital status (single, married), living arrangement (empty-nesters, non-empty-nesters), employment status (unemployed, agricultural work, non-agricultural work), and household income per capita (CNY). Economic status was estimated by household income per capita. It was divided into four types based on percentile including the first quartile (Q1), the second quartile (Q2), the third quartile (Q3), and the fourth quartile (Q4). Q1 was the poorest and Q4 was the richest. Here, the empty-nesters refers to those older adults with no children or whose children have already left home for at least six months, and they either live alone or with a spouse.

### ADL

We used the Activities of Daily Living Scale to assess older adult’s physical disability. The 14-item ADL score ranges from 14 to 56, with higher scores indicating the worse ability of daily living activities [36, 37]. The Cronbach’s α of the ADL was 0.80 in this study.

### Sleep quality

This study applied Pittsburgh Sleep Quality Index (PSQI) which consists of 19 item to evaluate sleep quality and disturbances during the past 30 days [38]. It is categorized into seven components: a) subjective sleep quality; b) sleep latency; c) sleep duration; d) habitual sleep efficiency; e) sleep disturbances; f) use of sleeping medication; g) daytime dysfunction. The PSQI score ranges from 0 to 21, with higher scores indicating poorer sleep quality. The Chinese version of this scale has high reliability and validity [39]. The Cronbach's α in this sample was 0.76.

### Psychological distress

This study used Kessler 10 (K10) to measure psychological distress. K10 is an effective tool for assessing mental health status and suitable for screening large-scale population mental illness. It can be defined a 5-point Likert scale and mainly focused on depression and anxiety during the past four weeks [40]. Its Chinese version has been proven to have good reliability and validity [41]. The Cronbach's α of the scale in this sample was 0.91.

### Suicidal ideation

Suicidal ideation was measured by the question “Have you ever seriously considered committing suicide?” We classified the affirmative respondents as having SI. This question was from the baseline National Comorbidity Survey (NCS) [42]. This measure of a single item from the National Comorbidity Survey has been widely used to assess SI in many previous studies [43–45].

### Statistical analysis

All statistical analyses were performed using Mplus 8.3 and SPSS 22.0. Firstly, we used descriptive analyses to describe the demographics. Secondly, Mann-Whitney U test and chi-square tests were used to compare the prevalence of SI across different subgroups. Thirdly, the relationships among the variables were examined by Spearman’s correlation analysis. Finally, Mplus 8.3 was used to test the hypothesized model, which analyzed the relationship between ADL and SI with mediating variables of sleep quality and psychological distress.

Our dependent variable is a binary variable, independent and mediating variables are continuous, so we used Bayesian estimation with uninformative priors which can learn more about parameter estimates and model fit [46]. The fit index for the model is Posterior Predictive *P*-Value (PP*P*). The posterior predictive checking compares the difference between actual data and the data produced by the hypothetical model [47]. An excellent-fitting model is expected to have a PP*P* around 0.5, which greater than 0.05 indicates an acceptable model fit. The reported credible intervals (CIs) were calculated at the 95% level and *P* values less than 0.05 were considered statistically significant.

Common method bias refers to the artificial covariation between the predictor variable and criterion variable caused by the same data source and measurement environment, which would seriously confuse the results. In the process of data collection, a standardized procedure was adopted, and a common method bias test was performed using Harman's single factor test [48]. The logic is based on that if common method variance is a problem, the first unrotated factor should account for a large proportion of the total variance, which was extracted from a factor analysis including all of items of this study. The results of the unrotated factors showed that the eigenvalues of 10 factors were greater than 1, suggesting that more than one factor underlies the data. The variance explained by the first factor was 20.43%, which was less than the threshold of 40%. This revealed that there was no serious common method bias variation.

### Ethical consideration

This study protocol was approved by the Ethical Committee of Shandong University School of Public Health. Written informed consents clarifying the study purposes, significance, methods, and risks were obtained from each participant.

## Supplementary Material

Supplementary Table 1
