# Supplementary material for: The relationship between activities of daily living and suicidal ideation among Chinese rural older adults: a multiple mediation model through sleep quality and psychological distress
Source: Aging (Albany NY). 2020 Nov 17;12(22):22614–25. doi: 10.18632/aging.103857 (PMC7746386; doi:10.18632/aging.103857)
Supplement: Supplementary Table 1 [file aging-12-103857-s001..pdf]

SUPPLEMENTARY TABLE

Supplementary Table 1 Multivariate logistic regression models for factors associated with suicidal ideation among the seniors in Shandong, China, 2019. (N=3,243).

Variables	Model 1 (Unadjusted)			Model 2 (Adjusted)		
	**OR**	**95% CI**	***P*-value**	**OR**	**95% CI**	***P*-value**
ADL	1.085	1.064-1.106	**<0.001**	1.037	1.010-1.061	**0.006**
Sleep quality	1.168	1.139-1.199	**<0.001**	1.039	1.006-1.071	**0.015**
Psychological distress	1.139	1.123-1.155	**<0.001**	1.117	1.097-1.134	**<0.001**
Controlling variables						
Gender (Male[Ref])				1.466	0.988-2.174	0.057
Age				0.966	0.944-0.989	**0.003**
Education (Illiterate[Ref])						
Primary school				1.018	0.768-1.349	0.903
Middle school or above				1.039	1.014-1.934	0.847
Marital status (Single[Ref])[a]						
Married				1.351	0.984-1.856	0.063
Living arrangement (Empty-nesters[Ref])[b]						
Non-empty-nesters				1.400	1.014-1.934	**0.041**
Employment status (Unemployed[Ref])						
Agricultural work				0.591	0.448-0.779	**<0.001**
Non-agricultural work				0.679	0.363-1.271	0.226
Household income per capita[c] (Q1[Ref])						
Q2				0.728	0.518-1.023	0.067
Q3				0.711	0.506-0.999	0.050
Q4				0.623	0.430-0.902	**0.012**
Cigarette smoking (No[Ref])						
Yes				0.666	0.457-0.971	**0.034**
Alcohol drinking (No[Ref])						
Yes				1.322	0.890-1.964	0.167
Physical exercise (No[Ref])						
Yes				0.650	0.502-0.841	**0.001**
R^2		0.228			0.259	

Notes: ADL= activities of daily living; [a] Singles include those who are unmarried (38, 1.17%), divorced (12, 0.37%) and widowed (778, 23.99%); [b] the empty-nesters elderly refers to those elderly with no children or whose children have already left home, and they either live alone or with a spouse; [c] Q1 was the poorest and Q4 was the richest.
